# The Malaysian Society of Toxicology: from establishment to evolution, a promising future!

**DOI:** 10.1186/s41021-023-00290-5

**Published:** 2023-12-11

**Authors:** Nurul Farahana Kamaludin, Firdaus Kamarulzaman, Rozaini Abdullah, Kok Meng Chan, Salmaan Hussain Inayat-Hussain

**Affiliations:** 1 Centre for Toxicology and Health Risk Studies (CORE), Faculty of Health Sciences, Universiti Kebangsaan Malaysia, Kuala Lumpur, Malaysia; 2 Natural Products Division, Forest Research Institute Malaysia, Kepong, Selangor Malaysia; 3 Department of Environmental & Occupational Health, Faculty of Medicine & Health Sciences, Universiti Putra Malaysia, Serdang, Selangor Malaysia; 4 Environment, Social Performance & Product Stewardship, Group Health, Safety & Environment, Kuala Lumpur City Center (KLCC), Petroliam Nasional Berhad (PETRONAS), Persiaran KLCC, Kuala Lumpur, Malaysia

**Keywords:** Toxicology, Malaysian Society of Toxicology, MySOT, Malaysia, SDGs

## Abstract

The Malaysian Society of Toxicology (MySOT), founded in 2010, emerged as a response to the growing need for a collective and interdisciplinary effort to study the effects of substances on human health, and the environment. By fostering collaboration between toxicologists, researchers, regulators, industry experts, and various relevant subject matter experts, MySOT has played a vital role in generating knowledge and promoting safety to safeguard both human and environmental well-being. Within the 13 years since its establishment, MySOT has made substantial progress in the advancement of toxicology in Malaysia. Over the years, MySOT has supported many initiatives, including organizing conferences, seminars, and workshops in which experts from various fields present their research, discuss emerging trends, and propose strategies to reduce toxic substance exposure risk. The society has also been actively involved in the broader landscape of toxicology research and policy influence in Malaysia. MySOT shoulders the responsibility of conveying accurate information and educating the public about health risks associated with toxic substances. Therefore, the society aims to collaborate with governmental organizations, professional bodies, and international toxicology organizations to share ideas, resources, and expertise. MySOT seeks to gather toxicological experts in the region and significantly contribute to a safer and healthier community, therefore supporting the United Nations Sustainable Development Goals (SDGs), by being actively involved with all of its stakeholders, both local and international.

## Introduction

The Malaysian Society of Toxicology (MySOT) founded in 2010 is currently progressing steadily on its rapid development as a professional society. It has expanded from a small group of Malaysian toxicologists and toxicology enthusiasts to a society with over 80 active members. Since its establishment, MySOT has steadfastly championed a range of transformative toxicology initiatives, organized a series of toxicology conferences, seminars, and workshops, and supported regional and global toxicology network. This article focuses on MySOT’s historical trajectory as well as on its aims for the future and ways to nurture more growth.

## From establishment to international participation

The concept of establishing a national society of toxicology was conceived in 1999 during a conversation between Dr. Salmaan Hussain Inayat-Hussain, who was then a junior lecturer at Universiti Kebangsaan Malaysia and Prof. Dr. Tetsuo Satoh, who was the Vice President of the International Union of Toxicology (IUTOX) at that time [1]. During the 3^rd^ International Congress of the Asia-Pacific Association of Medical Toxicology held in Penang, Malaysia, in 2001, a proposal for the formation of a society was put forth. However, during the Congress, the attendees, primarily comprised of medical doctors and pharmacists, suggested the establishment of a medical toxicology society, rather than a broader toxicology society encompassing all areas. Regrettably, the proposed plan did not advance beyond this point [1].

**Fig. 1:**
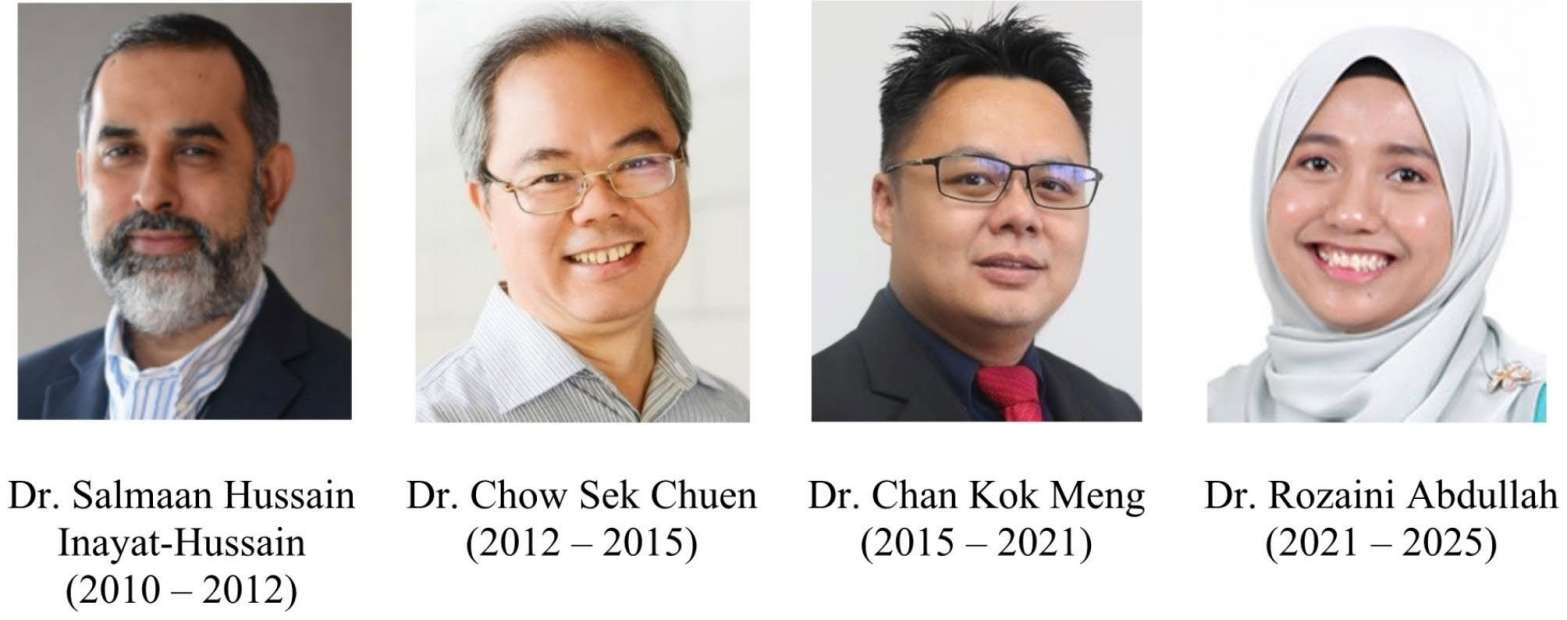
MySOT presidents and their respective duration from year 2010 to 2025

In 2009, Dr. Inayat-Hussain introduced the proposal during the COSTAM Pre-conference Toxicology Workshop in Kuala Lumpur, Malaysia. Consequently, this led to the establishment of a pro-tem committee for MySOT. Officially registered under the Registry of Societies of Malaysia (ROS) on 12 July 2010, MySOT had Dr. Inayat-Hussain, the organization’s founder, assuming the role of its inaugural president [1]. Dr. Chow Sek Chuen from Monash University Malaysia then held the presidency from 2012 to 2015, followed by Dr. Chan Kok Meng from Universiti Kebangsaan Malaysia from 2015 to 2021. Currently, Dr. Rozaini Abdullah from Universiti Putra Malaysia is the President, and she has been serving since 2021 (Fig. 1). Membership in MySOT is extended to scientists from both public and corporate entities, as well as students engaged in the field of toxicology, with inclusiveness not restricted to Malaysians. At present, MySOT has several active members hailing from Egypt, India, Indonesia, and Japan.

Since its inception, MySOT has been dedicated to increasing awareness and distributing information on toxicology in Malaysia. In pursuit of this goal, the society undertakes a range of activities organized to foster and sustain collaboration among governmental organizations, professional bodies, and individuals. Furthermore, MySOT actively fosters scientific research collaboration and facilitates the exchange of vital information among these stakeholders.

In addition, MySOT actively advances professional education in the subject of toxicology and engages in fruitful collaborations with international organizations. It was one of the pioneering member organizations to establish the Asia Quality Assurance Forum in 2013. Furthermore, MySOT is also a member of the WHO Chemical Risk Assessment Network, an international initiative that adopts a collaborative approach to evaluate potential dangers to human health [1].

**Fig. 2:**
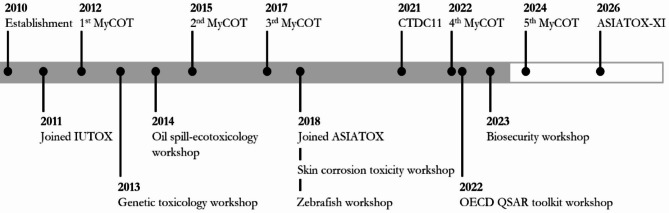
MySOT’s involvement in international societies and contributions to conferences and workshops

Since 2011, MySOT has maintained its membership within IUTOX, demonstrating its enduring commitment to the international toxicology community. Dr. Inayat-Hussain was elected as Director, IUTOX Executive Committee where he served for 2 terms from 2016 to 2022. MySOT has been given a second opportunity by the election of Dr. Chan Kok Meng, the advisor for MySOT, as Director of IUTOX Executive Committee in 2022 and is currently serving a three-year term. In a similar vein, 2018 saw the society’s accession to the Asian Society of Toxicology (ASIATOX), further fortifying its interconnectedness within the region. Figure 2 illustrates MySOT’s involvement as a national society with an international outreach by joining IUTOX and ASIATOX as well as organiser of where several conferences and workshops held from the year of its inception to 2026.

## Empowering professionals in toxicology

In accordance with its vision to be the scientific voice of toxicology in Malaysia, MySOT actively collaborates with various government agencies, industry, and professional groups to expand its national visibility and empower toxicologists in Malaysia. It also organizes various activities, including conferences and workshops as shown in Fig. 2. These events help unite local and international experts and encourage them to share their knowledge, present their research findings, and discuss emerging trends in toxicology. MySOT has collaborated with several research institutions, universities, and governmental bodies to promote toxicology-related initiatives. In this section, we will primarily highlight selected activities featuring the active participation of esteemed international speakers and participants.

## The Malaysian Congress of Toxicology (MyCOT)

**Fig. 3:**
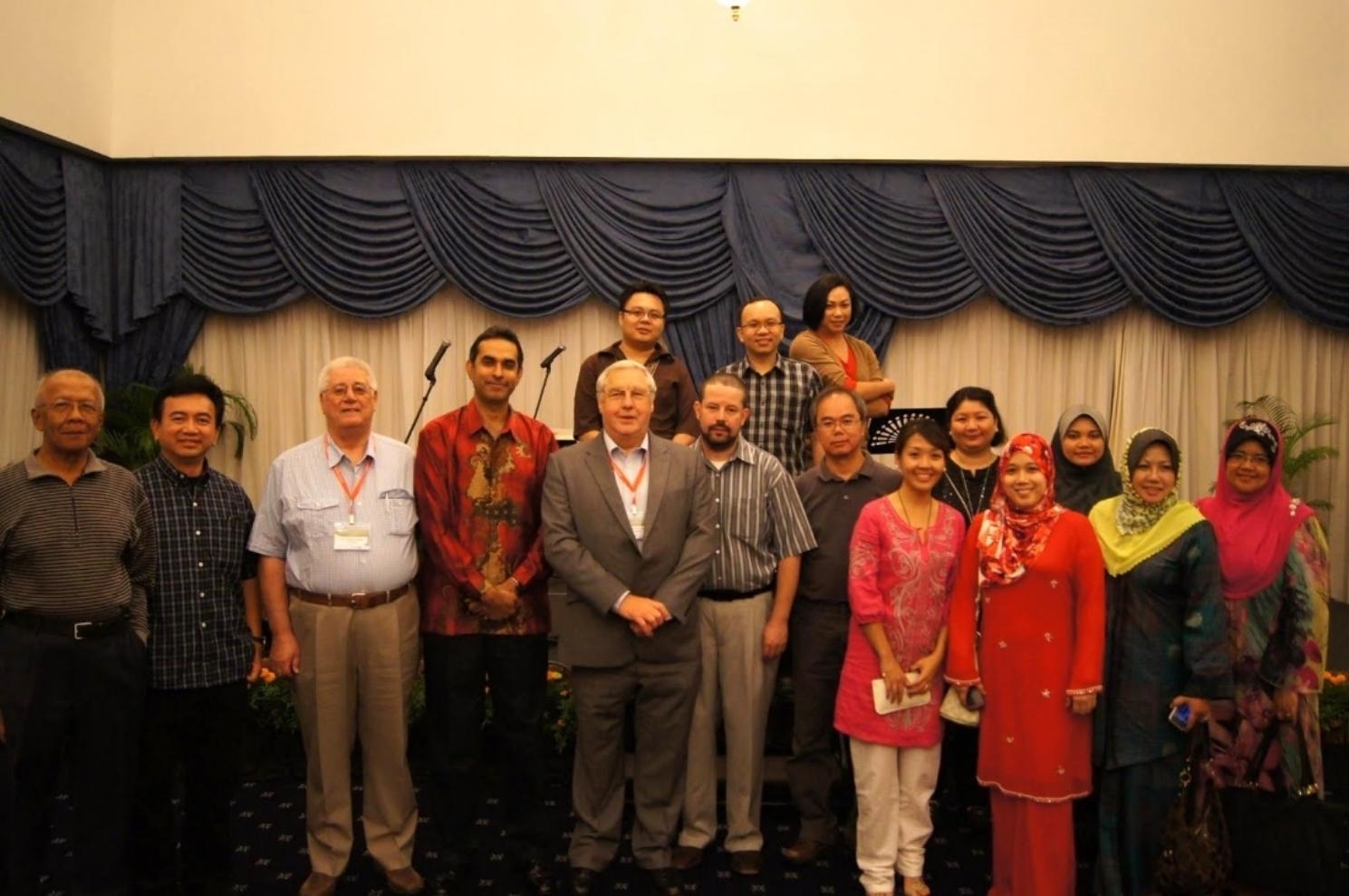
The 1^st^ MyCOT’s organizing committee with international speakers

**Fig. 4:**
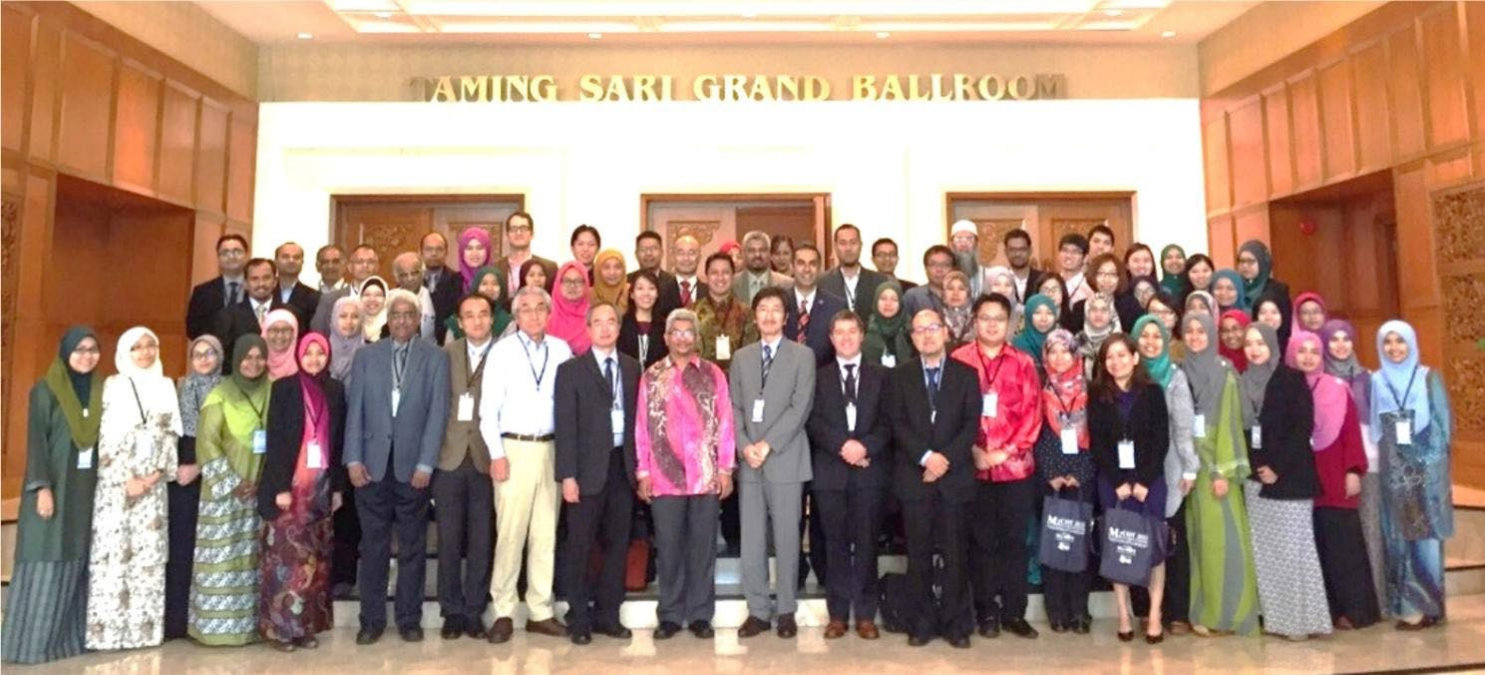
The 2^nd^ MyCOT held at The Royale Chulan Hotel, Kuala Lumpur, Malaysia in 2015

One of the most important agendas during its early years was organizing an international conference called the Malaysian Congress of Toxicology (MyCOT). The conference served as a crucial platform for MySOT to gain recognition, expand its network and forge future partnerships with toxicologists and other professionals at local and global levels. The first MyCOT, with the theme ‘From Mechanistic to Regulatory Toxicology’ (Table 1), was successfully held in 2012 at The Royale Chulan Hotel, Kuala Lumpur, Malaysia (Fig. 3). The congress was attended by local and international experts and stakeholders who presented their research and discussed various toxicology-related issues. MyCOT ultimately became a signature event for MySOT and was held with great success in 2015 (Figs. 4), 2017, and 2022.

**Table 1:** MyCOT with their respective year and theme

Event	Year	Theme
1^st^ Malaysian Congress of Toxicology	2012	From Mechanistic to Regulatory Toxicology
2^nd^ Malaysian Congress of Toxicology	2015	The Role of Toxicology Towards a Sustainable Society
3^rd^ Malaysian Congress of Toxicology	2017	Toxicological Advances in Shaping Sustainable Asian Communities
4^th^ Malaysian Congress of Toxicology	2022	Advances in Toxicology Post Covid-19 Disruption: Risk Assessment & Public Health Management

## The 11^th^ Congress on Toxicology in Developing Countries (CTDC) meeting

**Fig. 5:**
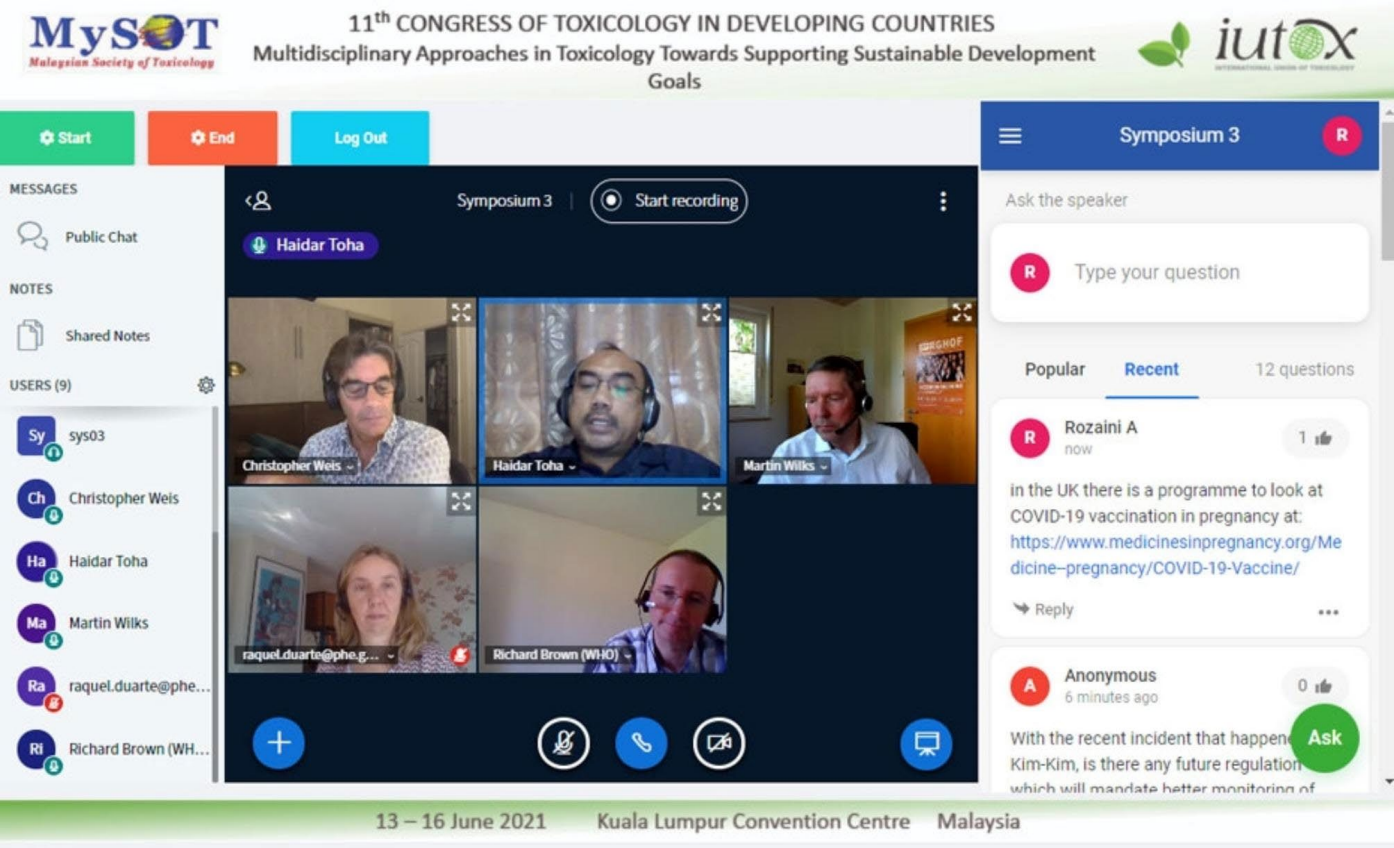
The 11^th^ CTDC meeting in 2021 was held as a fully virtual event due to the global COVID-19 pandemic

MySOT was honored to be given the exclusive opportunity by IUTOX to organize the 11^th^ CTDC meeting from 13 to 16 June 2021 with the theme of ‘Multidisciplinary Approaches in Toxicology Towards Supporting Sustainable Development Goals’. The meeting, which is held once every three years, provides an invaluable platform to discuss toxicological issues, with a primary focus on the crucial problems and challenges faced by developing countries and regions. The 11^th^ CTDC was initially planned as a physical meeting that had to be converted to a fully virtual event owing to the global COVID-19 pandemic (Fig. 5). Despite the hurdles, it attracted 260 delegates from 54 countries, 70% of whom were from developing countries.

## Toxicology-related workshops

**Fig. 6:**
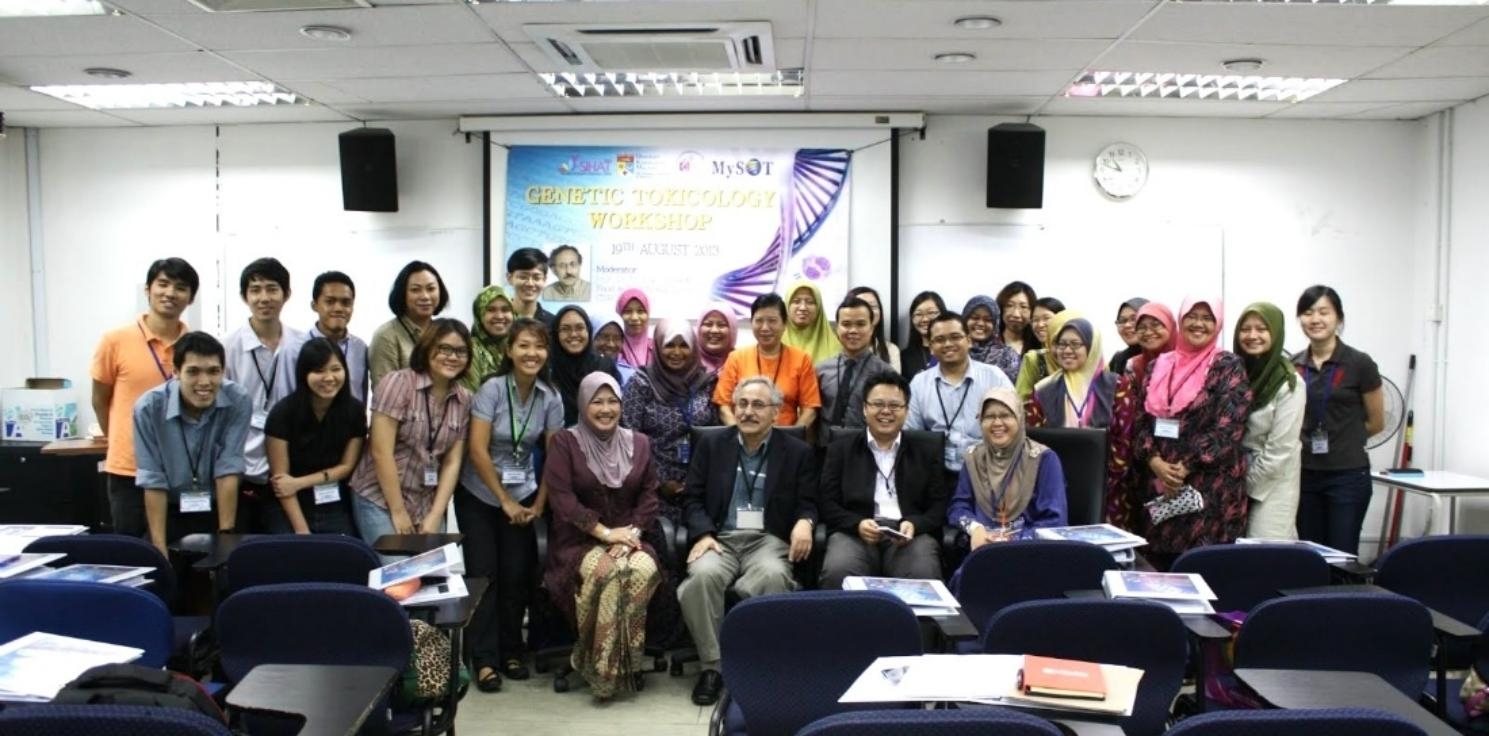
The participants of the Genetic Toxicology workshop with Prof. Dr. Michael Fenech

MySOT was also invited to conduct a genotoxicity workshop at the International Conference of Health Sciences organized by the Faculty of Health Sciences, Universiti Kebangsaan Malaysia in 2013. The primary purpose was to provide participants with a comprehensive understanding of genotoxicity and testing methodologies for assessing the risks associated with exposure to genotoxic substances (Fig. 6). Prof. Dr. Michael Fenech, a renowned genetic toxicology expert who developed the cytokinesis-block micronucleus (CBMN) assay, was the guest speaker. At present, this assay is an internationally accepted method for measuring DNA damage in human and animal cells. Academics, research officers, and postgraduate students from higher education institutions in Malaysia participated in the workshops.

**Fig. 7:**
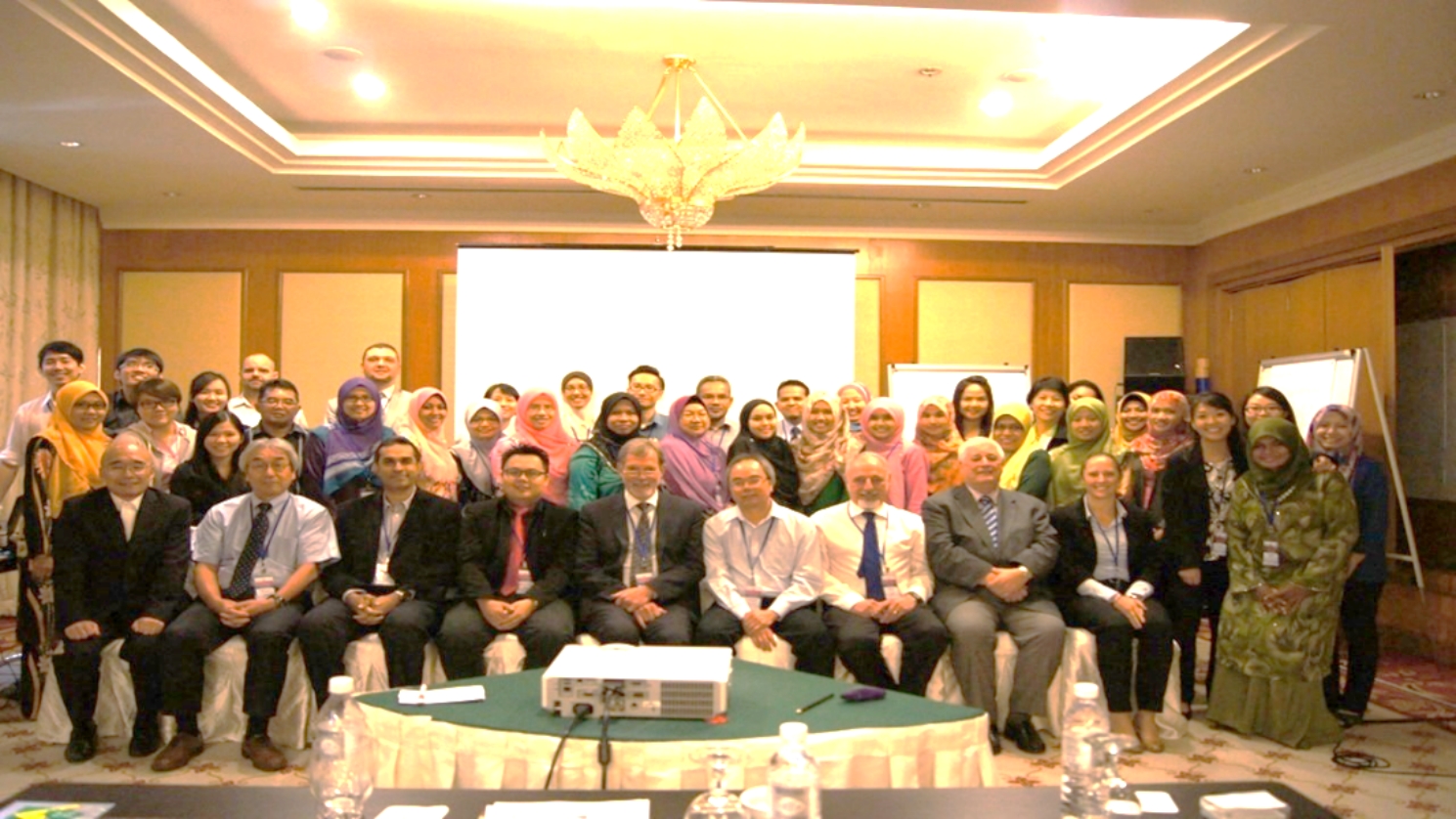
The participants and speakers of the Oil Spill Health and Ecotoxicological Effects and Risk Assessment Workshop conducted in Kuala Lumpur, Malaysia in 2014

Another major workshop that was successfully organized by MySOT in collaboration with the IUTOX was the Oil Spill Health and Ecotoxicological Effects and Risk Assessment Workshop conducted in Kuala Lumpur, Malaysia from 10 to 11 November 2014 (Fig. 7). Almost 50 participants comprising industry professionals and academics from various countries, including Malaysia, Japan, Australia, Thailand, Denmark, and England, were involved in the workshop.

**Fig. 8:**
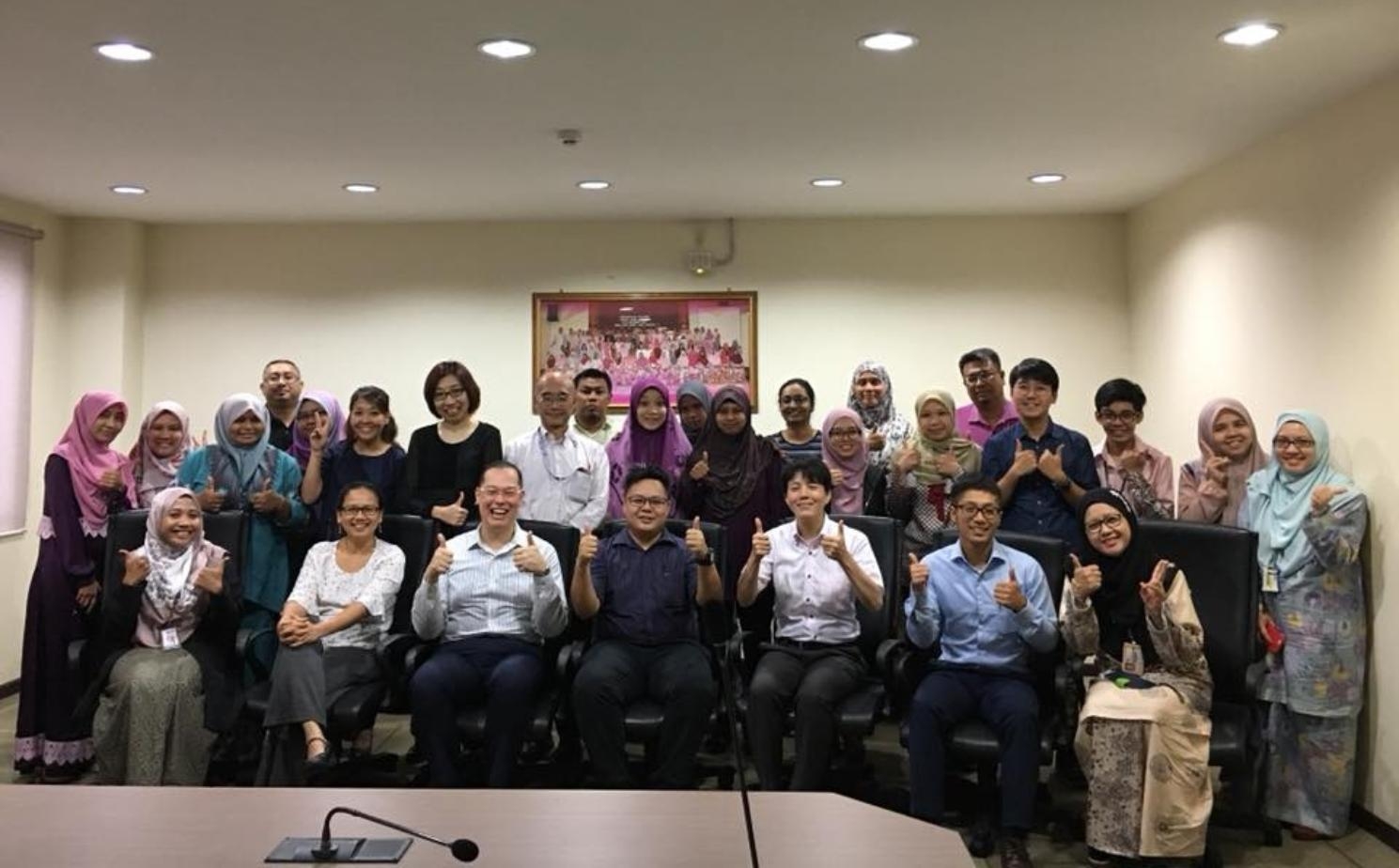
The speaker and participants of the Skin Corrosion Testing Demonstration Workshop

On 13 September 2018, Skin Corrosion Testing Demonstration Workshop was held at Toxicology Laboratory, Universiti Kebangsaan Malaysia (UKM), Kuala Lumpur, Malaysia (Fig. 8). This one-day workshop was jointly organized by MySOT, UKM, Prima Nexus and Japan Tissue Engineering Co., Ltd. (J-TEC). The workshop, which was attended by 30 participants, was focused on the basis physiology of skin, followed by the application of 3D reconstructed human epidermis model by J-TEC in skin corrosion testing (OECD Test 439). Dr. Alessandro Wataru Amici from J-TEC was the speaker of the workshop.

**Fig. 9:**
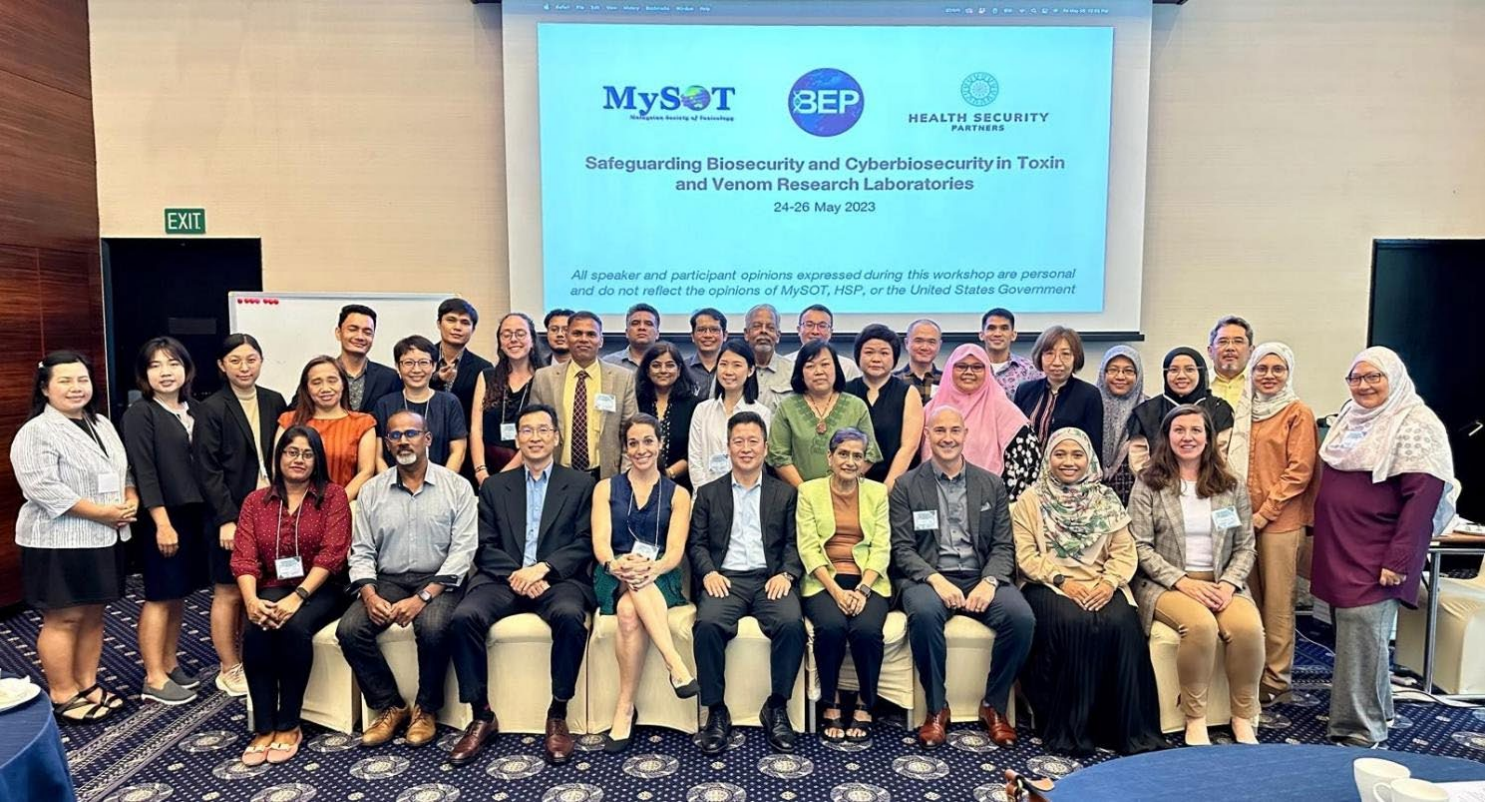
Participants and speakers of the Safeguarding Biosecurity and Cyberbiosecurity in Toxin and Venom Research Laboratories workshop in Singapore, 2023

From 24 to 26 May 2023, MySOT collaborated with Health Security Partners and the Biosecurity Engagement Program, USA to host a workshop on Safeguarding Biosecurity and Cyberbiosecurity in Toxin and Venom Research Laboratories (Fig. 9). The event, held in Singapore, brought together a diverse group of researchers from Malaysia, Singapore, and Thailand to discuss and share best practices to ensure the protection of materials, data and expertise in biosecurity and risk management. With great anticipation, MySOT looks forward to establishing collaborative partnerships with both regional and international experts to proactively address current issues and forthcoming challenges and find impactful solutions for a sustainable future in our communities and the environment.

## The 11^th^ International Congress of ASIATOX (ASIATOX-XI)

In 2026, MySOT will host the 11^th^ International Congress of ASIATOX (ASIATOX-XI) which will be held in Kuala Lumpur, Malaysia. MySOT has been nominated to host this important meeting by the ASIATOX society that fosters scientific cooperation, especially in the Asia region. The theme for the ASIATOX-XI meeting is ‘Advancements in Green Toxicology: Integrating Science, Sustainability, and Health’ aims to integrate the principles of green toxicology and sustainability, highlighting the imperative need to advance the field with a focus on environmental responsibility and long-term well-being. This theme aims to bring together toxicologists, researchers, industry experts, policymakers, and stakeholders from across Asia to share knowledge, exchange ideas, and collaborate on solutions that address the unique challenges and opportunities faced by the region. In addition, Dr. Abdullah who currently serves as the President of MySOT has been appointed as the President of ASIATOX for 2023 to 2026. This marks another significant milestone for MySOT in its contribution to toxicology.

## MySOT’s future direction

In the coming decade, MySOT aims to expand its role, improve services for its members, and make significant contributions to the nation. The organization seeks to attract a growing number of corporate professionals, enriching its membership with a diverse range of expertise and fostering collaboration between academia and industry. In pursuit of further scientific excellence, MySOT is exploring the possibility of collaborating with established journal publishers to facilitate the publication process for MySOT members, enabling them to disseminate their research findings and contribute to the wider scientific community more effectively. MySOT will also continue to actively collaborate with local and international governmental agencies, professional bodies, and toxicology organizations. Additionally, it aspires to introduce a certification program for toxicologists (i.e. Malaysian Registered Toxicologists) to recognize the professional credibility of individuals within this field, following similar practices in Asia [2].

## Conclusion

Within 13 years of its founding, MySOT has greatly contributed to the growth of toxicology in Malaysia and is steadily expanding to a higher level. It has provided platforms for collaboration and networking among toxicologists, leading to advancements in toxicology research and knowledge exchange that can ultimately benefit public health and safety. MySOT has also contributed to the advancement of knowledge and skills among Malaysian toxicologists and toxicology enthusiasts by organizing continuous training programs and workshops, thus improving the overall toxicology practice in Malaysia. The establishment of MySOT stands as a resounding testament to the unwavering dedication of toxicologists and experts in Malaysia to safeguard human health, the environment, and the prospects of future generations in the pursuit of a more sustainable world. Information on MySOT is available at MySOT official website (URL: www.mysot.org.my) and its official Facebook page (URL: www.facebook.com/MyS0T). Any inquiries should be addressed to mysot_secretary@yahoo.com.

## Abbreviations

ASIATOX: Asian Society of Toxicology
CBMN: Cytokinesis-block micronucleus
CORE: Centre for Toxicology and Health Risk Studies
COSTAM: Confederation of Scientific and Technological Associations in Malaysia
CTDC: Congress on Toxicology in Developing Countries
IUTOX: International Union of Toxicology
J-TEC: Japan Tissue Engineering Co., Ltd
KLCC: Kuala Lumpur City Center
MyCOT: Malaysian Congress of Toxicology
MySOT: Malaysian Society of Toxicology
OECD: Organisation for Economic Co-operation and Development
PETRONAS: Petroliam Nasional Berhad
QSAR: Quantitative structure-activity relationships
SDGs: United Nations Sustainable Development Goals
UKM: Universiti Kebangsaan Malaysia
